# Translation and validation of the Chinese version of the stroke knowledge test for the hypertension patient

**DOI:** 10.1038/s41598-023-44682-7

**Published:** 2023-10-14

**Authors:** Pao-Yu Wang, Yu-Wei Chen, Mei-Hsiang Lin

**Affiliations:** 1 Department of Nursing, MacKay Junior College of Medicine, Nursing and Management, New Taipei City, Taiwan, ROC; 2 Department of Neurology, Neuroscience Center, Landseed International Hospital, Taoyuan, Taiwan, ROC; 3 Department of Neurology, National Taiwan University Hospital, Taipei, Taiwan, ROC; 4 School of Nursing, National Taipei University of Nursing and Health Sciences, No. 365, Mingde Rd. Beitou Dist, Taipei, Taiwan, ROC

**Keywords:** Diseases, Health care, Neurology

## Abstract

The measurement of hypertensive patients’ stroke knowledge is an important stroke prevention indicator of health care service quality. The aim of this study was to develop a Chinese version of the Stroke Knowledge Test and examine its psychometric properties, reliability, and validity for hypertensive patients. A sample of 200 hypertensive patients completed the Chinese version of the Stroke Knowledge Test, and 30 of the participants were retested after 2 weeks. The final Chinese version of the Stroke Knowledge Test included 20 items with acceptable content validity (I-CVI = 0.88 ~ 1.00, S-CVI/Ave = 0.97). These items showed satisfactory internal reliability consistency (Kuder Richardson-20 = 0.62) and test–retest reliability was 0.77. The 40% of the difficulty index was in the acceptable range, and 25% was in the difficult range, as well as the 90% items overall values for discrimination were ranged from 0.2 to > 0.4. Known-group validity testing was performed based on the education level, and the result exhibited significant difference (F = 21.21, p < 0.001). The Chinese version of the Stroke Knowledge Test demonstrated acceptable psychometric properties, thus providing a new tool for the future care of hypertensive patients in Taiwan. It also could be as a reference for healthcare providers to educate hypertensive patients on stroke prevention.

## Introduction

Stroke is a global health and social problem and is one of the leading causes of death, dependency, and long-term disability in adults^1^. The burden of stroke is expected to increase in the coming years as global populations’ age^1,2^. This lack of knowledge of stroke risk factors and symptoms is a major public health challenge because stroke is common and may present as a medical emergency^2^. Patients with chronic diseases such as hypertension are at high risk of stroke^3^. Study has shown that blood pressure (BP) is the main determinant of ischemic stroke and intracranial hemorrhage. Controlling BP levels to < 150/90 mmHg reduces the risk of stroke^4^. In addition, empirical studies revealed that systolic blood pressure, diastolic blood pressure, pulse pressure, and mean arterial pressure could independently increase the risk of stroke^5^. Clinical studies have found that failure to provide accurate knowledge to hypertensive patients, or a patient’s lack of understanding of stroke, are associated with higher risk of stroke and complications^6^. Hypertension is the most common risk factor for stroke^4,7^. Furthermore, according to the survey of stroke prevention knowledge in hypertensive patients, who have sufficient understanding of stroke prevention, which can not only prevent the severity of stroke complications, but also prevent death^8^. However, survey in recent years showed that only 18.3% of hypertensive patients have good knowledge of stroke^7^.

Based on the above results, the researchers attribute the delay in treatment among hypertensive patients to their lack of awareness of stroke symptoms and available treatment options. In addition, several studies have shown that the community population with low levels of awareness and knowledge about stroke, including affected organs, causes, signs and symptoms, and treatment^2,9^. It is worth noting that providing information on how the public population behaves when they experience symptoms associated with stroke-related conditions^2^. Thus, measurement of the knowledge stroke and its risk factors in hypertensive patients can serve as a health care provider to inform the community to adopt stroke prevention behaviors^10^. Therefore, a valid and reliable assessment tool is crucial for determining whether hypertension patients have appropriate knowledge of stroke, and to improve clinical outcomes of long-term stroke^11^. Also, according to many systematic reviews of the relevant literature suggest that knowledge of stroke consists of an individual's understanding of the basic concepts of stroke, including its definition, epidemiology, risk factors, warning signs, emergency responses, symptoms, and treatment and recovery^12,13^.

Numerous instruments have been developed to measure stroke risk based on a patient’s behavior and actions ^14,15^. However, these instruments have limited utility in the assessment of patient stroke knowledge or their psychometric properties have not yet been validated^16^. Furthermore, the stroke knowledge of patients with hypertension is an important stroke-prevention indicator of healthcare service quality. The Stroke Knowledge Test (SKT) has been translated into various languages and adapted for use in different cultural contexts^3,17^. Strokes are known to be an important health problem in Taiwan. However, no valid and reliable scale is currently available to evaluate stroke knowledge in Taiwanese patients with hypertension. In addition, culture, which is highly specific and individual, has been considered as a variable in a series of studies dedicated to stroke knowledge^18,19^.

The success of translation of screening instruments depends on the achievement of equivalence, a difficult task given that no two languages are the same, both in the meaning of corresponding symbols and in the arrangement of symbols in phrases and sentences ^20^. According to Jones et al. ^21^ and Hui et al. ^21^, the cross-cultural translation process must take four equivalence considerations into account: (1) cultural equivalence: a similar meaning and relevance of the constructs being examined across cultures; (2) functional equivalence: the degree to which a concept performs the same way or elicits similar responses from individuals in two or more cultures; (3) item equivalence: the items are scored on the same scale for comparison; and (4) scalar equivalence: the items are measured in the same way. The aim of the present study was to develop the Chinese SKT (SKT-C) and verify its validity and reliability.

## Methods

### Study design and target population

For this study, we adopted a cross-sectional design and purposive sampling to survey patients at an outpatient cardiology department of a regional hospital in Taiwan. The inclusion criteria were as follows: (1) aged ≥ 20 years, (2) diagnosed with hypertension (average home blood pressure of 130/80 mmHg)^23^ or prescribed antihypertensive drugs by a specialist in hypertension and stroke (a co-investigator of this study), and (3) able to read and fill out questionnaires in Chinese. The exclusion criteria were having a mental illness, stroke, or dementia. The required sample size was estimated based on a 1:10 ratio of the number of questions in the questionnaire to the number of participants^24^. As the SKT contains 20 questions, 200 participants were recruited.

### Instrument

The SKT used in this study consists of 20 questions; the original version as a whole is considered one factor indicative of stroke knowledge ^25^. Each question has a single correct answer among five options, one of which is "do not know," which improves the validity of the questionnaire by preventing the participant from guessing the correct answer ^25,26^. One point is awarded for each correct answer, and no points are awarded for a wrong or "do not know" answer. The scores for the 20 questions are totaled, with a higher score indicating a greater degree of stroke knowledge^25^. The original scale’s content was selected based on a review of the literature as well as the original authors’ clinical experience of factors identified as important for the education of patients with stroke^25^. Items in the original scale cover the signs, symptoms, and risk factors of stroke ^27^, as well as factors related to the prevention, prevalence, and treatment of and recovery from stroke^25^. The original SKT has an internal consistency α value of 0.65 and a test-retest reliability r value of 0.82.

### Translation process

The translation and validation of the SKT-C was authorized by the authors of the original scale^25^. The translation was performed according to the Brislin guidelines^28^ and considering the four types of equivalence mentioned in the introduction ^21,22^. The translation was separately performed by two independent, bilingual individuals with a master's and doctoral degree in nursing, respectively. Each translator separately discussed their work with the investigators before finalizing their translation. Subsequently, two translators from English-speaking countries who understood Chinese and had never seen the original scale reverse-translated the Chinese version of the scale into English. Finally, the investigators invited the two translators and two reverse-translators to a group discussion, where they looked for any differences in semantics between the original scale and the translated scale (reflecting cultural and functional equivalence). The final version of the SKT-C retained all 20 items of the original scale (reflecting item equivalence), used the same scoring method as the original scale (reflecting scalar equivalence), and was tested for face validity^29^ with 10 patients recruited at the hypertension outpatient clinic. Finally, the SKT-C was fine-tuned according to the feedback received.

### Statistical analysis

Data collected in this study were processed using the SPSS version 23.0 software package. The correct answer rate and the “do not know” responses to the SKT-C were calculated as percentages, whereas expert validity and internal consistency of items were expressed as means and KR-20 values, respectively. The level of statistical significance was set at p < 0.05.

Content validity was evaluated by a panel of nine experts, including neurology specialists, nursing professors, and scholars proficient in scale construction, and the scale was revised and finalized according to their comments. The panel scored the scale items on content applicability and clarity. Each item was scored on a 1 to 5 scale, with higher scores indicating that a question is more appropriate. A score of 3 indicated that the item needed partial revision, while a score of 2 or lower suggested the item required substantial modification or deletion. The content validity index (CVI) was calculated by determining the proportion of items receiving a score of 4 or above^30^. The CVI value was calculated using the item-level CVI (I-CVI) and the average of the scale-level CVI (S-CVI/Ave)^31^.

We evaluated the properties of the items used to estimate stroke knowledge. The discrimination index and difficulty index were used to evaluate the discriminating ability of each item in the SKT-C^25,32^. For this evaluation, we sorted the participants’ stroke knowledge scores from highest to lowest. The upper group was defined as the top-scoring 27% of the total participants, whereas the lower group was defined as the lowest-scoring 27%^32^. The correct-answer rate of the upper and lower groups were calculated separately for each item of the scale. The difficulty index (P) was determined by summing the correct answer rate of the upper and lower groups and subsequently dividing that sum by the total number of participants in the two groups, expressed as a percentage. A higher difficulty index reflects an easier question. Items were categorized as follows: (a) P < 30% as difficult, (b) P = 30–70% as acceptable, and (c) P > 70% as easy^33,34^. The discrimination index (D) was determined by subtracting the correct-answer rate of the lower group from that of the upper group, multiplying the difference by two, and dividing that product by the total number of participants in the two groups. A higher discrimination index indicates a greater discriminating power. Items were categorized as follows: (a) D = 0–0.19 as poor discrimination; (b) D = 0.20–0.29 as acceptable discrimination; (c) D = 0.30–0.39 as good discrimination; and (d) D > 0.40 as excellent discrimination^33,34^.

The test-retest reliability of the SKT-C was measured using the Pearson correlation coefficient. Criterion-related validity was assessed using the known-group comparison method, in which statistically significant differences between the construct scores of two or multiple known groups indirectly verify the construct validity of the scale^35^. One-way analysis of variance (ANOVA) was used to compare differences in correct-answer rates of all items according to educational level to verify the construct validity of known-group differences. Known-group comparisons were performed to evaluate how well the scale can discriminate between participants enrolled in different groups, according to their educational level. Given the fact that the construct scores to be measured are known to vary substantially between certain groups, two or more groups can be deliberately selected for comparison. When the differences between groups are statistically significant, the construct validity of a scale is indirectly verified^35^. In previous studies, significant differences in stroke knowledge have been discovered among people with different educational levels^36^. Therefore, we expected that the correct-answer rate for the SKT-C would be lowest among those with only an elementary-level education.

### Ethical approval

The cross-sectional study was approved by the Institutional Review Board of Landseed International Hospital (IRB-19-014). All procedures performed in the studies were in accordance with the ethical standards of the institutional and with the 1964 Helsinki declaration. Informed consent was obtained from all individual participants in the study.

## Results

### Participant characteristics

A total of 200 patients with hypertension (mean ± standard deviation age: 56.2 ± 9.4 years) participated in this study. Most of them were high school graduates (n = 70, 35.0%), male (n = 120, 60.0%), and had not received any health education on stroke (n = 195, 97.5%) (Table 1).

**Table 1:** Demographic characteristics of the study sample (n = 200).

Characteristic	No. (%)	Mean ± SD	^†^F/p	Scheffe post hoc multiple comparison
Educational levels				
1. Elementary school	36 (18)	8.44 ± 2.41	21.21/ < 0.001	5 > 1 > 2 > 3
2. Middle school	26 (13)	8.96 ± 2.47		
3. High school	70 (35)	10.87 ± 2.41		
4.College	62 (31)	12.17 ± 2.45		
5.Graduate school	6 (3)	14.66 ± 1.03		
Age		56.2 ± 9.4		
Gender				
Male	120 (60)			
Female	80 (40)			
Received health education on stroke				
No	195 (97.5)			
Yes	5 (2.5)			

^†^The known-group validity of SKT-C (Chinese version of the Stroke Knowledge Test).

### Content validity

The S-CVI/Ave and I-CVI values of the translation were 0.99 and 0.88~1.00, respectively. For the evaluation of face validity, the 10 patients with hypertension completed the SKT-C in a range of 10~15 minutes. They stated that the questions were clear and easy to comprehend and suggested that the font size of the questionnaire should be increased.

### Estimation item properties.

Item difficulty was established by calculating the proportion of people obtaining the correct answer on an item^25^. Eight of the 20 SKT-C questions (40%; questions 1, 2, 4, 6, 8, 10, 11, and 16) had an acceptable level of difficulty, whereas five (25%; questions 3, 5, 14, 15, and 18) were too difficult and seven (35%; questions 7, 9, 12, 13, 17, 19, and 20) were very easy (Tables 2, 3). Two of the 20 SKT-C questions (10%; questions 14 and 18) had poor discrimination power, whereas the discrimination power of 12 (60%) was either acceptable or good (40% acceptable, and 20% good), and that of six (30%; questions 5, 6, 8, 10, 11, and 15) was excellent (Tables 2, 3). We modified those items that did not have an acceptable discrimination power or difficulty index. For example, question 14, "Approximately how many Australians are affected by stroke every year?" was eventually translated as "Among Taiwanese adults, what is the incidence of stroke per 1,000?" After several rounds of discussions, the final version of the SKT-C retained the same number of questions (20) as the original scale (Table 3).

**Table 2:** Classification of questions according to the difficulty index (P) and discrimination index (D).

Difficulty index (P)		
Interpretation	Items n (%)	Difficulty index (mean ± SD)
Difficult (< 30%)	5(25)	18.26 ± 8.95
Acceptable (30–70%)	8(40)	54.39 ± 13.4
Easy (> 70%)	7(35)	79.15 ± 5.72
Discrimination index (D)		
Interpretation	Items n (%)	Discrimination index (mean ± SD)
Poor (0–0.19)	2(10)	0.099 ± 0.03
Acceptable (0.2– 0.29)	8(40)	0.23 ± 0.03
Good (0.3– 0.39)	4(20)	0.35 ± 0.02
Excellent (> 0.4)	6(30)	0.57 ± 0.14

**Table 3:** Item analysis of the Chinese version of Stroke Knowledge Test^#^ (n = 200).

Item wording	Total (%)	Upper (n = 58)	Lower (n = 65)	Difficulty index (%)	Discrimination index (%)
1.The most common type of stroke occurs when	61.5	74.1	46.2	60.2	0.27
^†^2. Which of the following significantly increases the risk of stroke?	37.5	51.7	29.2	40.5	0.22
^†^3. Is atrial fibrillation (AF) a type of irregular heartbeat that could increase stroke risk?	16	41.4	4.6	23.0	0.36
4. Which age group is more at risk of stroke?	31.5	48.3	20	34.2	0.28
5. The warning signs of transient ischemic attack (TIA) disappear?	18	43.1	3.1	23.1	0.40
6. Which of the following is a warning sign of stroke?	71	94.8	44.6	69.7	0.50
7. For someone who has had a stroke, the main purpose of rehabilitation is to	90.5	100	80.7	89.1	0.20
8.Taking aspirin assists in preventing stroke by	43	79.3	7.7	43.5	0.71
9.You are at greater risk of stroke if	85	89.9	69.8	79.9	0.20
^†^10.What could potentially happen in the event of a transient ischaemic attack (TIA)?	56.5	91.4	20	55.7	0.71
11. Surgery can sometimes help to prevent another stroke by	63	96.6	29.2	62.9	0.67
12.What method of treatment is available for people who have had a stroke?	80	96.6	60	78.3	0.36
13.The most important known risk factor for stroke is	76.5	91.4	53.8	72.6	0.37
^†^14. In Taiwanese adults, what is the incidence of stroke per 1,000?	4.5	10.3	3.1	6.7	0.07
^†^15. What is the increase in the risk of stroke in those who consume alcohol excessively compared to non-drinkers?	35	50.6	4.6	27.6	0.46
16. Which of the following is an example of a physical disability caused by stroke	68.5	84.5	52.3	68.4	0.32
17. To reduce the risk of stroke, you need to	83.5	96.6	67.7	82.2	0.28
^†^18. What is the increase in the risk of stroke in those who smoke 20 cigarettes per day compared to non-smokers?	8.5	17.2	4.6	10.9	0.12
19. If someone has symptoms suspected of stroke, when should you ring for an ambulance?	64	82.8	63.1	72.5	0.20
20. Rehabilitation can assist someone who has suffered	81.5	89.7	69.2	79.5	0.20

^#^Correct responses (%). ^†^slight adjustments were made in the choice of words and semantics to account for cultural differences.

### Clinical validity (known‑group comparisons)

The One Way ANOVA showed that the total SKT-C score was the highest for participants with the graduate-level education. Scheffe's post-hoc test for multiple comparisons showed that participants with elementary school education had the lowest SKT-C scores. Participants with postgraduate education scored the highest. The analysis of differences in known groups supported that the SKT-C has good construct validity (Table 1).

### Reliability

The overall KR-20 of SKT-C was 0.62. The test-retest reliability coefficient was 0.77 (p < 0.0001).

## Discussion

In this study, the SKT-C was developed via translation and validation for the measurement of the stroke knowledge of patients with hypertension, providing a new instrument for the future care of such patients in Taiwan. It may also serve as a reference for healthcare providers to educate patients with hypertension on stroke prevention. The scale was translated into Chinese by using a back-and-forth translation method. The translation process was carried out following the guidelines put forward by Jones et al.^21^ to ensure linguistic accuracy that reflects local culture. Most participants were willing to answer all the questions, and the time to complete the questionnaire was considered reasonable. In summary, the scale is fit for clinical use.

In line with a previous study^30^, rigorous testing revealed that the SKT-C was reliable and effective. Expert validity is a type of content validity^30^, and experts from different fields, including neurologists and scholars in nursing education, were invited to contribute to this study. Their input in modifying the questionnaire improved the understanding among local participants and reduced the rate of random answers. In addition, the panel of experts suggested that question 14 be modified to assess the knowledge of the incidence of stroke among adults in Taiwan, to achieve the objective of testing stroke knowledge that is in line with local conditions. The I-CVI score ranged from 0.88 to 1.00, and the S-CVI/Ave score was 0.99, which is similar to that of the Malaysian version of the Stroke Knowledge Scale translated by Sowtali et al.^3^, and indicates good content validity^37,38^. Furthermore, the face validity test^39^ conducted in this study ensured that participants correctly understood the questions of the assessment tool, which improved the credibility of the evaluation results.

The quality of a constructed knowledge scale can be determined using the difficulty index and the discrimination index of test questions^40^. Based on the total number of correct answers, we divided participants into upper- and lower-scoring groups to further calculate the ability of each item to discriminate between high and low scorers. Sixty percent of the SKT-C questions had an acceptable or good discrimination index (0.20–0.39), and thirty percent exhibited an excellent discrimination power (> 0.40). This result was better than that of a similar study by Sowtali et al.^3^, in which 75% of the items in the Malay version of the SKT had only an acceptable discrimination power. Another result of this study was that 40% of the items had an acceptable difficulty index, and 35% were easy, in contrast with the results by Sowtali et al.^3^, in which 70% of the items had an acceptable difficulty and only 5% were easy. A possible explanation for this discrepancy is that the educational level of the participants differed between the populations. Most of the participants in the present study had graduated from college or high school, whereas most of those in the Malay study had graduated only from high school or below. Graduates have more opportunities to learn and could benefit more from consultations than patients with lower levels of education^30^. Therefore, stroke knowledge can reasonably be affected by educational attainment. In the present study, the one-way ANOVA revealed that the total SKT-C score was the highest for participants with a graduate-level education, followed by those with college, high school, middle school, and elementary school-level education. Scheffe's post-hoc test for multiple comparisons showed that participants with elementary school education had the lowest SKT-C scores, followed by those with middle school education. Participants with postgraduate education scored the highest. The analysis of differences in known groups indicated that the SKT-C has good construct validity. Pearson analysis revealed a high correlation (*r* = 0.77; p < 0.0001). The demonstration that the SKT has adequate temporal stability can be interpreted as a further indication of the construct validity of the SKT-C.

Along with this issue, two questions, which assessed the causes and the incidence of stroke among Taiwanese people, had a poor discrimination index and a high difficulty index. Similar questions in the Malaysian version SKT scale and the original SKT scale were also considered difficult, and attracted the most "do not know" answers^3,25^. Further analyses revealed that most participants lacked stroke knowledge^3^. Therefore, the SKT-C retained items with a poor difficulty index or a poor discrimination index to facilitate the assessment of stroke knowledge among respondents from different cultures. This type of knowledge should also be strengthened in the course of promoting stroke awareness in the future. The overall KR-20 of the SKT-C was 0.62, which indicate acceptable reliability^35^ and is similar to the SKT results reported by Sullivan et al^25^. The test-retest reliability coefficient correlation was significant and > 0.7 which established its test-retest reliability. Although the test-retest reliability values were less than those obtained for the original SKT scale (ie, = 0.82) ^25^, nevertheless, the values obtained from this study were in the recommended acceptable range^35^. Therefore, the results of this study showed that the SKT-C demonstrated acceptable psychometric properties.

### Strengths and limitations of the study

This lack of knowledge of stroke risk factors is a major public health challenge. Moreover, hypertension is the most common risk factor for stroke. The strength of our study is that patients were recruited from a community population sample, which will help the scale to strengthen prevention services for patients in the community. Furthermore, the study used different reliability and validity tests to test the reliability and validity of the instrument. Despite its strengths, there were several limitations of this study. This study was a cross-sectional study, and purposive sampling method, this study only recruited hypertensive patients at one regional hospital in Taiwan. The study results may be biased due to regional differences, so the findings cannot be generalized to hypertensive patients in other regions of Taiwan. Additionally, a discriminant validity test was not performed; therefore, the construct validity of SKT-C should be further examined. Despite the limitations, this is the first study to validate the Chinese version of the SKT in a sample using a community population of hypertension patients in Taiwan. The SKT-C has acceptable internal consistency reliability and validity. It is a suitable assessment tool for evaluating stroke knowledge in hypertensive patients in Taiwan.

## Conclusion

The SKT-C underwent rigorous translation, revision, and validation processes. This study verified that the SKT-C has good content validity and acceptable item analysis results, it can be used as a stroke prevention assessment instrument for community population hypertensive patients, ensuring that healthcare professionals address the health needs of hypertensive patients, thereby improving the quality of care.

## Data Availability

All data analyzed during this study are included in this published article.

## References

[1] Kim J (2020). Global stroke statistics 2019. Int. J. Stroke..

[2] Soto-Cámara R (2020). Knowledge on signs and risk factors in stroke patients. J. Clin. Med..

[3] Sowtali SN (2016). Translation and validation of the Malay version of the stroke knowledge test. J. Arrhythm..

[4] Wajngarten M, Silva GS (2019). Hypertension and stroke: Update on treatment. Eur. Cardiol..

[5] Hägg-Holmberg S (2019). The role of blood pressure in risk of ischemic and hemorrhagic stroke in type 1 diabetes. Cardiovasc. Diabetol..

[6] Droste DW (2014). Stroke awareness in luxemburg: Deficit concerning symptoms and risk factors. Clin. Med. Insights..

[7] Abate AT, Bayu NH, Mariam TG (2019). Hypertensive patients' knowledge of risk factors and warning signs of stroke at Felege Hiwot Referral Hospital, Northwest Ethiopia: A cross-sectional study. Neurol. Res. Int..

[8] Tibebu NS (2021). Knowledge on prevention of stroke and its associated factors among hypertensive patients at debre tabor general hospital: An institution-based cross-sectional study. Risk Manag. Healthc. Policy..

[9] Kharbach A (2020). Level of knowledge on stroke and associated factors: A cross-sectional study at primary health care centers in Morocco. Ann. Glob. Health.

[10] Owolabi MO (2022). Primary stroke prevention worldwide: Translating evidence into action. Lancet Public Health..

[11] Grech R, Grech P (2021). The stroke knowledge assessment tool (SKAT): Development, reliability and validity. J. Med. Health Sci..

[12] Koton S (2013). Burden and outcome of prevalent ischemic brain disease in a national acute stroke registry. Stroke..

[13] Krzystanek E (2020). Adequate knowledge of stroke symptoms, risk factors, and necessary actions in the general population of southern Poland. Brain Sci..

[14] Centers Centers for Disease Control and Prevention (CDC). *Behavioral Risk Factor Surveillance System Survey Questionnaire.* Atlanta, GA: Department of Health and Human Services, Centers for Disease Control and Prevention (2001)

[15] Billings-Gagliardi S, Mazor KM (2005). Development and validation of the stroke action test. Stroke..

[16] Hou WH (2017). A systematic review of tests assessing stroke knowledge. J. Cardiovasc. Nurs..

[17] Chakroun-Walha O (2021). Stroke knowledge among emergency centre visitors: A cross-sectional multicenter survey. Afr. J. Emerg. Med..

[18] Grech P, Grech R (2020). Stroke knowledge: Developing a framework for data integration in a sequential exploratory mixed method study. Res. Methods Med. Health Sci..

[19] Yap KH (2021). Understandings of stroke in rural Malaysia: Ethnographic insights. Disability Rehab..

[20] Nida E, Venuti L (1964). Principles of correspondence. The Translation Studies Reader.

[21] Jones PS (2001). An adaptation of Brislin’s translation model for crosscultural research. Nurs. Res..

[22] Hui CH, Triandis HC (1985). Measurement in cross-cultural psychology: A review and comparison of strategies. J. Cross Cult. Psychol..

[23] Wang TD (2022). 2022 guidelines of the Taiwan society of cardiology and the Taiwan hypertension society for the management of hypertension. Acta Cardiol. Sin..

[24] DeVon HA (2007). A psychometric toolbox for testing validity and reliability. J. Nurs. Scholarsh..

[25] Sullivanm K, Dunton NJ (2004). Development and validation of the stroke knowledge test. Top Stroke Rehabil..

[26] DeVellis RF (2017). Scale development theory and applications.

[27] Van Veenendaal H, Grinspun DR, Adriaanse HP (1996). Educational needs of stroke survivors and their family members, as perceived by themselves and by health professionals. Patient Educ. Couns..

[28] Brislin RW (1970). Back-translation for cross-cultural research. J. Cross Cult. Psychol..

[29] Waltz CF, Strickland OL, Lenz ER (2016). Measurement in nursing and health research.

[30] Lynn MR (1986). Determination and quantification of content validity. Nurs. Res..

[31] Polit DF, Beck CT, Owen SV (2007). Is the CVI an acceptable indicator of content validity?. Appraisal and recommendations. Res. Nurs. Health..

[32] Mitra NK (2009). The levels of difficulty and discrimination indices in Type a multiple choice questions of pre-clinical semester 1 multidisciplinary summative Tests. IeJSME..

[33] Tejinder S, Piyush G, Daljit S (2009). Principles of Medical Education.

[34] Ananthakrishnan N, Ananthakrishnan N, Sethuraman KR, Kumar S (2000). Item analysis-Validation and banking of MCQs. Medical education, Principles and practice.

[35] Portney LG, Watkins MP (2000). Foundations of clinical research: Applications to practice.

[36] Umar AB (2019). Stroke knowledge among middle and high school students. J. Int. Med. Res..

[37] Polit DF, Beck CT (2009). Essentials of Nursing Research: Appraising Evidence for Nursing Practice.

[38] Polit DF, Beck CT (2006). The content validity index: Are you sure you know what ’ s being reported? Critique and recommendations. Res. Nurs. Health..

[39] Mosier CI (1947). A critical examination of the concepts of face validity. Educ. Psychol. Meas..

[40] Naqvi AA (2020). Cross-culture adaptation and validation of English version of Rheumatoid Arthritis Knowledge Assessment Scale (RAKAS) in patients with rheumatoid arthritis. Int. J. Rheum. Dis..

